# The Rulers of South London

**Published:** 1899-11-25

**Authors:** 


### THE RULERS OF SOUTH LONDON.

In view of the high death-rate which has long prevailed in the parish of St. George-the-Martyr, Southwark, every little improvement in that deadly district should be matter for congratulation. Lately 1,419 boxes of human remains, probably representing twice that number of bodies, have been removed from the crypt of St. George's Church. This ought to do some good. We are afraid, however, that some of the rulers of this district fail to see the advantage of sanitary improvements. They love their dear old smells. According to the report of the vestry meeting in the *South London Press*, one of the vestrymen, "with tears in his voice, declared that not only had poor people been condemned to pay a twopenny rate, but the ashes of the sacred dead had been disturbed." The septic dead would perhaps have been a more accurate description. Another bewailed the 4s. 2d. which he had had to pay to take away dead bodies. "The four-and-twopence of which he had been robbed would have bought his kiddies boots, and his blood boiled when he saw the well-paid medical officer sitting there and knew that he was responsible for this robbery." This is the sort of thing that a medical officer in a London parish, in the very centre of knowledge, has to put up with when he does his duty. The climax came when a vestryman expressed his indignation that Dr. Waldo should actually have been paid a fee for superintending the work, on which more than one voice cried out, "That accounts for it." If anyone thinks that London affairs are managed even with ordinary decency let him read a few of the reports of the meetings of the vestry of St. George-the-Martyr.

